# Migraine and cluster headache – the common link

**DOI:** 10.1186/s10194-018-0909-4

**Published:** 2018-09-21

**Authors:** Anne Luise Vollesen, Silvia Benemei, Francesca Cortese, Alejandro Labastida-Ramírez, Francesca Marchese, Lanfranco Pellesi, Michele Romoli, Messoud Ashina, Christian Lampl, on behalf of the School of Advanced Studies of the European Headache Federation (EHF-SAS)

**Affiliations:** 1 Danish Headache Center and Department of Neurology, Rigshospitalet Glostrup, Faculty of Health and Medical Sciences, University of Copenhagen, Copenhagen, Denmark; 2 Health Sciences Department, University of Florence and Headache Centre, Careggi University Hospital, Florence, Italy; 3 Department of Medico-Surgical Sciences and Biotechnologies, Sapienza, University of Rome, Polo Pontino, Latina, Italy; 4 Dep Internal Medicine, Division of Vascular Pharmacology, Erasmus Medical Center, Rotterdam, The Netherlands; 5 Child Neuropsichiatry Unit, University of Palermo, Palermo, Italy; 6 Medical Toxicology, Headache and Drug Abuse Center, University of Modena and Reggio Emilia, Modena, Italy; 7 Neurology Clinic, University of Perugia - S.M. Misericordiae Hospital, Perugia, Italy; 8 Department of Neurogeriatric Medicine, Headache Medical Center Linz, Ordensklinikum Linz Barmherzige Schwestern, Seilerstaette 4, 4010 Linz, Austria

**Keywords:** Migraine, Cluster headache, Calcitonin gene-related peptide (CGRP), Anti-CGRP (receptor) monoclonal antibodies – mAbs, Neuromodulation, Trigeminovascular system, Hypothalamus

## Abstract

Although clinically distinguishable, migraine and cluster headache share prominent features such as unilateral pain, common pharmacological triggers such glyceryl trinitrate, histamine, calcitonin gene-related peptide (CGRP) and response to triptans and neuromodulation. Recent data also suggest efficacy of anti CGRP monoclonal antibodies in both migraine and cluster headache. While exact mechanisms behind both disorders remain to be fully understood, the trigeminovascular system represents one possible common pathophysiological pathway and network of both disorders. Here, we review past and current literature shedding light on similarities and differences in phenotype, heritability, pathophysiology, imaging findings and treatment options of migraine and cluster headache. A continued focus on their shared pathophysiological pathways may be important in paving future treatment avenues that could benefit both migraine and cluster headache patients.

## Background

In the field of cephalalgias, migraine has a prominent role (35,311 publications retrieved for search terms “migraine” in PubMed, accessed on August 15, 2018), with the recent breakthrough in therapeutics, represented by the successful clinical development of calcitonin gene-related peptide (CGRP) antibodies [1]. However, in the last 40 years, the number of papers published yearly for cluster headache (CH) has been constantly increasing (3845 publications retrieved for search terms “cluster headache” in PubMed, accessed on August 15, 2018), and new evidence is accumulating about epidemiology, including gender issues, pathophysiology and imaging. Differences and similarities between the two cephalalgias are presented here with a comparative approach. The clinical continuum that unexpectedly but not infrequently characterizes migraine and CH patients increases the value of such a comparison between the two diseases.

## Epidemiology and genetics in migraine and cluster headache

Migraine is a highly prevalent disease, affecting at least 12% of the general population [2], with a lifetime prevalence up to 25% among women [3]. CH is a primary headache disease with an estimated prevalence at 0.5–1.0/1000 of the general population [4]. Both migraine and CH can be present from childhood and their prevalence increases until nearly 40 years of age, after which it gradually decreases [3, 5]. Twin studies demonstrate a heritability around 42% for migraine [6]. Five concordant monozygotic twin pairs with CH have been reported [7], indicating the importance of genetic factors in both disorders. The risk of first-degree relatives of patients with CH to develop CH is between five and fifteen times greater than that of the general population [7]. However, CH does not exhibit a clearly recognizable inheritance pattern. The genetic background of CH has been an unexplored field for years; genetic studies have been performed only recently, in small numbers of patients or as case reports. To date, targeted genes have been investigated, including the calcium voltage-gated channel subunit alpha1 A (CACNA1A) [8], three nitric oxide synthase (NOS) genes [9], the period circadian regulator 3 (PER3) [10] and the hypocretin receptor 2 (HCRTR2) [11], and none showed evidence of involvement in CH. In some families, the mode of inheritance is likely to be autosomal dominant with incomplete penetrance; in other families, it is more likely to be multifactorial or autosomal recessive [12, 13] (see Table 1).

**Table 1:** Epidemiological and genetic similarities and differences in migraine and cluster headache

	Migraine	Cluster headache	
Epidemiology and genetics	Predominant in adulthood [3, 5]	Predominant in adulthood [3, 5]	Similarities
	Familial aggregation [6]	Familial aggregation [7]	
	12-15% of the general population [2]	0.5-1 ‰ of the general population [4]	Differences
	The first-degree relatives of patients have a 3-fold increase of migraine headache, compared with the general population [14]	The risk of first-degree relatives of patients to develop CH is between five and fifteen times greater than that of the general population [7]	
Supposed mode of inheritance	Polygenic disorder and rare monogenic variants [15]	Autosomal dominant with incomplete penetrance, multifactorial or autosomal recessive [12, 13]	

In migraine, first-degree relatives of patients have a 3-fold increase in migraine, compared to the general population [14]. The risk increases in migraine with typical aura, supporting the idea that distinct genetic factors may regulate the inheritance of specific forms of migraine [15]. Rare monogenic migraine subtypes can be caused by precise genetic mutations, as in the case of familial hemiplegic migraine; a rare genetic disorder with dominant autosomal transmission due to mutations of three main genes (CACNA1A, ATP1A2 and the sodium channel 1 A SCN1A) [16]. These genes are not involved in common migraine [17] or in CH [8], in which numerous genes and environmental factors contribute to susceptibility in a manner still unclear. Several studies have failed to identify any association between genetic variants and common forms of migraine indicating that autosomal-dominant inheritance is unlikely, unless the penetrance of the gene is very low. Migraine is currently considered a polygenic disorder: multiple predisposing genes contribute, each with a small effect size, to the underlying risk [16]. New gene alterations have recently been related to CH [18–20], and a large meta-analysis has mapped 38 distinct genomic loci expressed in vascular and smooth muscle tissues, associated with migraine [21]. These results should be furthered in larger populations. Although both diseases are characterized by family aggregation, most noticeable in adulthood, CH is a rare disease, with a stronger genetic influence. Accordingly, the mode of inheritance is likely to be different between migraine and CH, and whether some genetic traits are shared between the two disorders is unknown.

## Pathophysiology

In the pathophysiology of migraine and CH, both the peripheral nervous system and central nervous system are involved. Three key structures interact and subsequently involve cortical areas as well: the trigeminovascular system, parasympathetic nerve fibers (trigeminal autonomic reflex) and the hypothalamus [22].

### Trigeminovascular system and trigemino-cervical reflex

In migraine and CH, pain is likely due to activation of the trigeminovascular system [22]. Nociceptive nerve fibers originate from the trigeminal ganglion (TG) and reach intracranial structures such as the dural, arachnoid and pial blood vessels, cerebral arteries and extracranial structures [22–25]. From TG the nociceptive signals project to neurons in the trigeminal cervical complex (TCC), including the trigeminal nucleus caudalis (TNC), and the dorsal horn of the upper cervical spinal cord (C1-C2) [24–27]. These projections from the TCC terminate on neurons of the trigeminal brainstem nuclear complex [28] and transmit all somatosensory information via further projections: to thalamic neurons (via a trigemino-thalamic tract), to hypothalamic nuclei (via a trigemino-hypothalamic tract), to basal ganglia nuclei and to brainstem nuclei including the locus coeruleus (LC) and periaqueductal gray (PAG) [25, 26, 28–30]. Subsequently, these structures reach several cortical areas involved in processing aspects of the nociceptive signals [26, 30].

#### Neuroimaging and neurophysiological investigations

Various neuroimaging studies implicate the brainstem in the pathophysiology of migraine and CH. In migraine, abnormalities are seen in both ascending and descending nociceptive pathways during ictal and inter-ictal phases [31]. Positron emission tomography (PET) imaging studies showed increased dorsal pons activation in migraine patients during the ictal phase [32]. Functional magnetic resonance imaging (fMRI) studies reported increased functional connectivity between the cortical and subcortical regions involved in nociceptive processing and the PAG [33, 34], having connections coming from the thalamus, hypothalamus, and autonomic nervous system [31].

A dysfunction of pain control systems in both headaches and a role of the brainstem in their pathogenesis is also supported by neurophysiological studies. In migraine, loss of habituation, lower cortical pre-activation and abnormal sensitization was seen [35]. In CH, altered pain perception and decreased pain thresholds was found [36].

In migraine some studies reported that the blink reflex (which reflects excitability of interneurons in the brainstem) is delayed and reduced in amplitude [37, 38]. However, other studies did not confirm these conclusions [39, 40]. In cluster headache patients, during active phase, and on the headache side, a pronounced lack of habituation of the brainstem and a general sensitization of pain processing is seen [41]. These results point towards dysfunctional connections between the brainstem and trigeminovascular system, again supporting the trigeminovascular hypothesis [38].

In summary, electrophysiological studies show that the migraine brain presents some interrelated functional characteristics: 1. lack of habituation of evoked responses to repeated stimuli; 2. cortical dysexcitability**.** The lack of habituation was reported by examining visual evoked potentials (VEP) [41–50] by using magneto-electroencephalography [51, 52], with somatosensory [45, 46] and auditory [53, 54] evoked cortical potentials pain (laser, LEP) [55] and event-related (contingent negative variation) responses [56, 57] in migraine between attacks [58]. Regarding cortical dysexcitability, conflicting results were presented in various trials, suggesting cortical hypoexcitability [59, 60] as well as hyperexcitability [61, 62]. Recent works suggest that abnormal rhythmic activity between thalamus and cortex induce a low level of cortical preactivation. This might explain the abnormal functional characteristics in migraine mentioned above. Abnormal processing could be due to hypoactivity of some pathways (such as the serotonergic pathway), causing heightened response to repeated stimuli, thus resulting in an excessive energy demand [63]. Changes in energy demand may disrupt cerebral metabolic homeostasis and thus activate the major alarm signalling system of the brain, the trigeminovascular system, ultimately resulting in a migraine attack [63].

### Trigeminal autonomic reflex and cranial parasympathetic symptoms

Somatosensory pathways are connected to autonomic pathways through reflex connections from the TNC to the superior salivatory nucleus (SuS). The SuS contains neurons that are part of the cranial parasympathetic autonomic vasodilator pathway [28, 64, 65]. These neurons project to the cranial vasculature, including dura mater, to the nasal and oral mucosa and lacrimal glands mainly through the sphenopalatine ganglion (SPG) [28]. Activation of the cranial SuS-parasympathetic pathway is believed to directly contribute to cranial autonomic symptoms found in cluster headache and up to 50% in migraine patients [29, 66]. Indeed, activation of this pathway induces a dilation of intracranial vessels and a cascade of events that results in plasma protein extravasation, neuropeptide release from dural vascular terminals of post-SPG neurons [28], local dural release of inflammatory mediators with perivascular alteration and activation and sensitization of the trigeminovascular system [23, 27]. The SuS also has a bidirectional connection with the hypothalamus (including the lateral [65, 67], paraventricular, dorsomedial and pre-optic hypothalamic nuclei [65, 68]), as well as with the limbic and cortical areas [65].

### Hypothalamus

The hypothalamus is involved in numerous physiological functions including controlling circadian rhythm [22, 69]. Furthermore, it has several connections involved in pain modulation in migraine as well as in cluster headache [36]. The hypothalamus also partakes in autonomic and endocrine regulation [23]. Preclinical data show that specific hypothalamic nuclei, such as the paraventricular and lateral hypothalamus, reach the TNC and SuS neurons through descending projections [22, 65, 67, 68, 70, 71], thus influencing and triggering somatosensory and autonomic neurovascular mechanisms [23]. The premonitory symptoms of headaches are considered the clinical side of an underlying hypothalamic dysregulation. Many neuro-endocrinological data support the hypothesis of hypothalamic–pituitary–adrenal axis failure in these primary headache disorders [72].

#### Neuroimaging and neurophysiological investigations

fMRI studies report a role of the hypothalamus in pain modulation during the pre-ictal phase of attacks in migraine patients. Particularly, it is hypothesized that the anterior part of the hypothalamus may be involved in migraine chronification, whereas the posterior part may play a role in the acute pain phase [73].

In CH, activation in the hypothalamic grey matter ipsilateral to the side of a headache during attacks is seen with PET [74] and fMRI [75]. Also, altered functional connectivity of the hypothalamus and anterior thalamus were described. A voxel-based morphometry (VBM) study [64] revealed concomitant grey matter volume increase of this hypothalamic region, but other VBM studies did not substantiate these results [76–79]. Interestingly, a recent work [80] hypothesized that the anterior hypothalamus might contribute to the circadian rhythm of CH attacks [22], whereas the posterior part might generate the restlessness experienced by CH patients during the attack [81].

Alterations of resting state activity [82], were found in the attention network ipsilateral to the pain and in the contralateral cerebellar network. This result coincides with previous repetitive transcranial magnetic stimulation (rTMS) studies showing increased cortical excitability ipsilateral to the pain in CH [82], similar to that in a migraine [83]. Resting state studies showed altered activity of the medial frontal cortex which is part of various resting state networks important in pain perception [75, 84]. This disorganized connectivity could be a consequence of white matter microstructural alteration described in CH [85].

Lastly, cognitive processing studies employing event-related potentials are useful in elucidating cortical activation time courses during cognitive processing [86, 87]. The hypothalamic dysfunction might also explain the habituation deficit of the brainstem and the general sensitization of pain processing detected in patients with CH [88]. Neurophysiological studies of sensory evoked potentials show various abnormalities but not as homogenously as displayed in migraine [89–91]. The intensity dependence of auditory evoked potentials is also increased in CH patients, during and outside active phase, possibly suggesting decreased serotoninergic activity in the hypothalamic pathways [92].

### Other brain structures

In addition to the above-mentioned studies involving brainstem and hypothalamus, patients with primary headaches experience dynamic structural [93] and functional [75] changes in cortical-subcortical areas involved in nociception.

In migraine, fMRI and resting-state fMRI studies show marked abnormalities both ictally and interictally in areas involved in nociceptive processing and networks involved in mediating cognitive, attentional, somatosensory and emotional components of pain [33, 52, 94–97], respectively. These networks may influence multisensory integration and pain experience in migraine patients. Structural MRI studies also show a decrease in grey matter in various regions of the brain such as frontal, parietal and temporal cortex (Table 2). However, neuroimaging data on the association of white matter hyperintensities and migraine has been conflicting. Some studies show a higher occurrence of subcortical, deep, and cerebellar ischemic hyperintensities in migraineurs [98], whereas other studies fail to confirm such findings [99].

**Table 2:** Structural and Functional abnormalities in migraine and cluster headache

	Migraine	Cluster headache
Structural MRI (VBM/DTI)	Decreased Grey Matter in: frontal lobes, prefrontal cortex, left medial prefrontal (MPFC), brainstem cortex, cerebellum, temporal lobes Right Superior Temporal; bilateral insula; cingulated cortex; orbitofrontal cortex right occipital lobe right posterior parietal cortex [226–230] Reduced Fractional Anisotropy values: superior frontal lobe; medial frontal lobe; Right Inferior Frontal [227, 230] Thickening of the cortical mantle in the Somatosensory cortex [231]	Decrease Grey Matter in: right thalamus, bilateral posterior, Hypothalamus, right posterior cingulate cortex, left inferior parietal lobe, head of the right caudate nucleus, bilateral middle frontal gyrus, right-middle temporal gyrus, right precentral gyrus, left insula [64, 78, 79] Increased Grey matter: right cuneus [78] Grey matter volume changes: Temporal lobe, hippocampus, insular cortex, cerebellum [77]. Changes in Fractional Anisotropy: Brainstem, thalamus, internal capsule, superior and inferior temporal region, frontal lobe, occipital lobe and cerebellum [101] Cortical thinning was found in the contralateral angular and precentral gyrus contralateral to the headache side [232]
fMRI	Enhanced activation in: perigenual part of anterior cingulate cortex [233] red nucleus, substantia nigra [234], dorsolateral pons [24, 235], cerebellum, insula, cingulate, prefrontal cortices, anterior temporal pole, hippocampus [32] Decreased activation in: somatosensory cortex [233]	Enhanced activation in: Posterior hypothalamus, anterior and posterior cingulate cortex, thalamus, basal ganglia, cerebellar hemispheres, prefrontal, insular and temporal cortices [64, 74, 95]
Structural MRI (VBM/DTI)	Stronger functional connectivity: • periaqueductal grey to the ventrolateral prefrontal cortex, supramarginal gyrus, anterior insula, precentral gyrus, postcentral gyrus, and thalamus [33] • anterior cingulate cortex to middle temporal, orbitofrontal cortex, and dorsolateral prefrontal cortex [229] • caudate nucleus to the parahippocampal gyrus, amygdala, insular cortex, and putamen; nucleus accumbens to the parahippocampal, anterior cingulate cortex, orbitofrontal cortex [236] • Medial prefrontal cortex to the posterior cingulate cortex (coppola 2017) • Medial prefrontal cortex and left to the right inferior parietal lobules and bilateral insula [237] Atypical Functional connectivity of: • salience network, default mode network, central-executive network, somatomotor network, and frontoparietal attention network [34] • left rostral anterior cingulated cortex, bilateral prefrontal cortex and right thalamus [97]	Stronger functional connectivity of: • hypothalamus to parts of the frontal, parietal and temporal cortex during headache free intervals; to the Anterior Cingulate Cortex and Posterior Cingulate Cortex during the acute spontaneous CH [87] • attention network ipsilateral to a headache paine and in the contralateral cerebellar network [75] Atypical Functional connectivity of: • the hypothalamus both ipsilateral and contralateral to the CH side and the salience network [238] Decreased functional connectivity of: • the hypothalamus with the medial frontal gyrus, precuneus and cerebellar areas [97]

*VBM* voxel based morphometry, *DTI* Diffusion Tensor Imaging

In CH, a decrease of the grey matter in several regions was shown using structural MRI [78]. Structural alterations in the striatum [93, 100] and atrophy of the thalamus and the caudate nucleus has also been reported. Importantly, in addition to a decrease also an increase in the right cuneus was observed [78]. Recent resting-state fMRI studies found abnormal functional connectivity in the sensorimotor and primary visual networks during the pain-free period, as well as between the hypothalamus and pain network areas in active phase [84, 87, 95] (Table 2). Diffusion-tensor imaging studies investigating white matter microstructural changes offer contradictory findings [36, 78, 101]. Some report the absence of white matter abnormalities [78]. Others report widespread white matter microstructural changes, particularly in the pain networks such as the frontal lobe, parietal lobe, temporal lobe and thalamus [36, 85].

## Clinical picture

### Phenotypes

Migraine and CH are diagnosed according to the International Classification of Headache Disorders (ICHD-3), which are evidence-based primarily on patient populations [102]. Although the clinical presentation of migraine and CH is usually different, these primary headaches often share some similarities in the headache phenotype, such as unilateral and severe pain and some associated symptoms including aura [103, 104] (Table 3). Moreover, coexistence of these two primary headaches simultaneously has been reported [105].

**Table 3:** Clinical similarities and differences amongst cluster headache, migraine without aura and migraine with aura

Headache phenotype	Cluster Headache	Migraine without aura	Migraine with aura	
Location	Strictly unilateral	Usually unilateral		Similarities
Intensity	Severe/very severe	Moderate/severe		
Associated symptoms	Nausea, photophobia and phonophobia			
	Aura (≈20%) [103]	-	Aura	
Quality	Excruciating, stabbing	Deep, pulsating		Differences
Duration	15-180 minutes	4-72 hours		
Radiation	Orbital, supraorbital and/or temporal	Frontotemporal		
Circadian/circannual rhythms	Nocturnal [22] Spring/autumn	Early morning [108]-		
Frequency	Once every other day to eight times a day	Once every other day		
Most common triggers	Alcohol [5]	Stress, cycling female hormones [239], [113] (but also alcohol)		
Aggravators	-	Routine physical activity		
Cranial autonomic symptoms	Ipsilateral, prominent	Bilateral, mild [66]		
Disability during headache	Restlessness or agitation	Severe impairment or require bed rest		

CH attacks are usually associated with multiple prominent ipsilateral cranial autonomic symptom, such as conjunctival injection, lacrimation, rhinorrhea, forehead sweating, miosis and/or ptosis [22, 106]. These symptoms have also been described in migraineurs, but patients usually report only one symptom (forehead sweating the most frequent) and in contrast to CH, they are less frequent, bilateral and mild [66].

Interestingly, different cohorts have revealed that CH patients without comorbid migraine frequently experience accompanying ‘migrainous associated symptoms’, such as photophobia, phonophobia, nausea or vomiting [104, 107]. Furthermore, CH attacks are associated with specific chronobiological features, mainly circadian (most frequently nocturnal) and circannual (most frequently spring or autumn) rhythms [22]. In contrast, migraine attacks are most frequently reported to occur during the day and no clear seasonal rhythm has been stablished yet [108].

When migraine attacks occur on 15 or more days/month for more than three months it is considered chronic [102]. Each year 2.5–3% of patients with episodic migraine transform to chronic migraine (CM), fortunately these patients frequently revert back to episodic migraine [109, 110].

CH attacks occurring for one year or longer without remission or with remission periods lasting less than three months (10–15%) are classify as chronic [102]. CCH may be unremitting from onset (de novo), or evolve from the episodic type and in some patients a change from chronic to episodic may occur [111]. Furthermore, a recent consensus from the European Headache Federation defined refractory CCH as a situation that fulfills ICHD-3 for CCH with at least three severe attacks per week despite at least three consecutive trials of adequate preventive treatments [112].

### Triggers

Migraine and CH patients report a remarkable number of common triggers – both naturally occurring events such as stress, sleep, alcohol intake and weather changes [106, 107, 113], but also pharmacological triggers [22, 114]. It has been suggested that these triggers are common trigeminal system activators [105, 109].

Identification and avoidance of attack triggers plays an important role in management of patients with migraine and CH. Attack triggers may also provide clues to their underlying pathophysiology [115]. While naturally occurring attack triggers are useful in management of individual patients they may be of limited use in experimental provocation studies. Thus, in a study of self-reported triggers of migraine with aura only 17% of patients developed an attack following exposure to their natural attack trigger [116]. For a comprehensive review on specific natural attack triggers of primary headaches see Pellegrino et al. 2017 [115].

The earliest pharmacological provocation studies in migraine and CH patients explored histamine [117–119] and found that histamine infusion, which causes endogenous nitric oxide (NO) formation, induces attacks in both migraine and CH. In a double-blind, randomized pretreatment study in 20 migraine patients without aura (MwoA) [117], a 20 min intravenous histamine infusion was pretreated with mepyramine (0.5 μg/kg/min for 10 min) or placebo infusion (*n* = 10, each). In the placebo pretreated group 7 of 10 MwoA patients reported migraine-like attack following histamine infusion compared to 0 of 10 in the mepyramine group. In the placebo pretreated group the average time to peak headache was 5 h. In CH, nine patients received subcutaneous injection of histamine (0.01 mg/kg body weight) [120]. All nine CH patients developed CH-like attacks after a median time of 45 min. The study was neither blinded nor placebo controlled.

Glyceryl trinitrate (GTN), a prodrug of NO, was given intravenously (0.5 μg/kg/min for 20 min) in a double-blind, placebo controlled, cross over study on 12 MO patients [121]. At a median time of 5.5 h after GTN infusion 8 of 10 patients fulfilled criteria for a migraine attack compared to only one after placebo. In CH, several non-placebo controlled provocation studies found that GTN induces CH in episodic active phase in 33–100% of patients [122–125] and in CCH in 20–78% of patients [125, 126]. In remission phase episodic CH patients GTN induced no attacks [122–124]. Mean time to onset of attacks was 12–72 min after infusion start [120, 122, 124]. NO, among other things, increases intracellular cyclic guanosine monophosphate (cGMP) [127]. Sildenafil, a phosphodiesterase 5 inhibitor, which also increases intracellular cGMP, also induced migraine attacks in 10 of 12 MO patients compared to 2 of 12 after placebo [128]. In CH, cases of sildenafil (prescribed for erectile dysfunction) triggering CH attacks in active phase [129, 130] and even triggering an active phase itself [131] have been reported. In a randomized, double-blind, placebo controlled cross-over study in 12 MwoA patients, vasoactive signaling molecule CGRP was infused intravenously (2.0 μg/min for 20 min) [132]. In the paper, authors stated that three of nine MO patients developed delayed migraine attacks as strictly defined by criteria from the International Headache Society on CGRP compared to zero of nine on placebo. When revisiting these results and applying newer, modified criteria for pharmacologically induced migraine-like attacks, CGRP induced delayed migraine-like attacks in six of nine MO patients compared to one in nine after placebo [133]. In a recent study, 32 CH patients (9 episodic active phase, 9 episodic remission phase, and 14 chronic) received intravenous infusion of CGRP (1.5 μg/min for 20 min) or placebo in a randomized, double-blind, placebo controlled cross-over study [134]. CGRP induced cluster-like attacks in 89% of episodic active phase patients compared to 11% after placebo, and in 50% of chronic patients compared to 0% after placebo. In episodic remission phase CH patients neither CGRP nor placebo induced any attacks. Median time to onset of attacks was 20 min in CCH and 30 min in episodic active phase CH. This was the first placebo controlled provocation study in CH. Authors conclude that these findings point to the possibility of efficacy of CGRP antagonism, already known to prevent and abort migraine [135–138], in CH as well. Such antibodies against CGRP are currently under investigation in CH [22]. Recently the efficacy in reduction of weekly attacks in episodic but not CCH was announced [139].

Thus, although migraine and CH have several pharmacological triggers in common, the time to onset of attacks seems to vary predictably between the two diseases with CH generally being triggered faster than the average induced migraine attack [117, 120, 132, 140, 141]. In migraine, delayed attacks are thought to arise from the pharmacological trigger playing a role relatively early in spontaneous migraine attack initiation [114]. Thus, the short time to attack in CH might reflect a shorter cascade of events in CH attack initiation relative to migraine.

Migraine and CH are linked pathophysiologically by common neuronal structures, however, they are (usually) influenced differently by lifestyle, environmental, hormonal and genetic factors [107]. This shared pathophysiology is supported by common environmental and pharmacological attack triggers and similar efficacy with some treatments (see next section). Unfortunately, research about the pathophysiological interactions between diseases is scarce and these questions remain to be elucidated.

### Gender aspects

Migraine and CH show distinct and inverse gender-related characteristics. Migraine is two to three times more common in women than in men, estimates varying from 13% to 17% for females and 7.6% to 10% for males [142]. On the contrary, CH is a male-dominated disorder with the ratio of men to women estimated to range from 3:1 to 7:1 [143]. Puberty is a turning point for the predominance of gender in both primary headaches, which in childhood show a similar distribution by gender [144, 145]. According to the onset of the disease, gender differences are more evident in the third decade of life for both migraine and CH, and women with CH show a further peak of incidence between the ages of fifty and sixty years [143]. In older people, the gender-related aspects vanish in both disorders.

Women experience migraine or CH differently than men. Women report more severe and longer attacks [146]. Moreover, women with migraine are more likely to report nausea, vomiting, photophobia, phonophobia and aura associated with headache [147]. Men and women with CH have similar clinical phenotypes [148], with no apparent differences in pain intensity, quality and location. Women with CH report more nausea and vomiting than men, but it is unclear whether this is caused by a generally higher proportion of concomitant migraine [149]. Additionally, women with CH appear to respond more poorly to some abortive and preventative treatments [150]. The reasons for the opposite gender characteristics in migraine and CH are not fully understood. The underlying causes are likely to be multifactorial, involving both biological and psychosocial factors. Among biological factors, previous studies have focused on fluctuations in sex hormones and the exploration of genetic factors, without obtaining a definitive response [151].

## Treatment

Migraine and CH therapy includes the acute therapy to abort the single attack, and preventive therapy to reduce attack frequency, duration and severity and the use of acute headache medications.

### Acute therapy

As in migraine, CH attacks respond well to acute therapy with triptans [152–154]. Nevertheless, differently from migraine, the oral route of administration is not usually recommended in CH, because of the delayed effect compared to subcutaneous or intranasal administration. On the other hand, acetaminophen and non-steroidal anti-inflammatory drugs are only used in the acute therapy of migraine and not in CH [155]. Shared pathophysiological mechanisms as reviewed in previous sections could explain the efficacy of triptans in both diseases.

Another acute approach for the treatment of CH attacks is inhalation of 100% oxygen through a face mask (with a flow of 12–15 l/min). Interestingly, a recent randomized placebo-controlled clinical trial on 22 patients reported that high-flow oxygen was significantly more effective than air in the acute treatment of migraine attacks [156], and it has been suggested that this treatment could have greater responses in migraine patients with cranial autonomic symptoms [157] or migraine-cluster and cluster-migraine variants (these rare phenotypes are not included in the ICHD-3). An inhibition of activated trigeminal nociceptive afferents or the autonomic pathway could be one of the mechanisms explaining its efficacy in both migraine and CH [158].

Lastly, in patients affected by CH, when oxygen and triptans are ineffective, intranasal lidocaine (sprayed in the ipsilateral nostril) should be considered [125]. Clinical trials provided conflicting data about its efficacy in migraine [159–161].

Taken together, the previous suggests that, although with different preferable route of administration (for triptans) and response rate (for oxygen inhalation) migraine and CH share responsiveness to some acute strategies (see Table 4).

**Table 4:** Efficacy of acute therapies in migraine and cluster headache

	Migraine	Cluster headache
NSAIDs	Effective [155]	Not effective
Triptans	Oral route of administration	Subcutaneous sumatriptan/ intranasal sumatriptan or zolmitriptan [153, 154] effective
Inhalatory oxygen	Effective in about 46% of patients [156]	Effective: about two-third of patients [240]
Intranasal lidocaine	Conflicting data [159, 160]	Effective [125, 241]

### Preventive therapy

Different drug categories are effective in the prophylactic treatment of patients affected by episodic or CCH, even though, unlike in migraine, few randomized clinical trials have been conducted [162]. Similarities and differences in migraine and CH preventive therapies are summarized in Table 5.

**Table 5:** Efficacy of preventive therapies in migraine and cluster headache

	Migraine	Cluster headache
Verapamil	Effective [242]	Effective in high-dose (360 up to 960 mg)
Litihum	No large RCTs; ineffective in small trials; efficacy clues in “cyclic migraine” [169]	Effective [166]
Steriods	Reduced recurrence of attacks in patients coming to emergency department [171]	Effective (usual dosage ≥40 mg)
Antiepileptic drugs	Effective	Efficacy clues in open uncontrolled studies, not confirmed by RCTs.
GON blockade	Effective in chronic migraine [173]	Effective [172, 243]
Melatonin	3 mg per day are effective [186]	10 mg per day are effective [185]

High-dose verapamil is the most frequently used in CH preventive therapy [163]. Interestingly, few studies suggested the efficacy of verapamil in migraine prophylaxis [164, 165]. Lithium carbonate is mainly used as a prophylactic drug in CCH to reduce the attack frequency in patients [166, 167]. To date, no randomized clinical trials have studied the efficacy of lithium in migraine prophylaxis. Small open trials reported conflicting results in migraine [168, 169]. A short term effective therapy for CH is represented by prednisone [77, 170] which can be used for short-duration episodes or to induce a rapid remission (usually within 3–10 days). Evidences about the use of steroids in the preventive therapy of migraine do not allow precise conclusions. Nevertheless a recent review showed that steroids demonstrated a good efficacy in reducing the recurrence of migraine in patients visiting the emergency department for acute attacks [171]. Blockade of the greater occipital nerve (GON) ipsilateral to the pain, with injection of corticosteroids and local anesthetic is effective in CH [172] and was also shown to be effective in treatment of CM [173].

In migraine, the efficacy of sodium valproate and topiramate has been documented in RCTs [174, 175]. In CH, even though open uncontrolled studies indicated a good efficacy, RCTs did not show any clinical efficacy of sodium valproate and topiramate [176–180].

Open trials showed clinical efficacy of local injection of onabotulinumtoxin A into the sphenopalatine ganglion (SPG) both in CH [181] and in refractory CM therapy [181]. The Phase III REsearch Evaluating Migraine Prophylaxis Therapy 1 and 2 (PREEMPT 1 and 2) have shown the efficacy of Onabotulinumtoxin A in reduction of headache days in CM, using a specific injection protocol [182, 183]. The PREEMPT study protocol was also used in a 28 week, open-label trial, with refractory CCH [184]. A more than 50% reduction in headache minutes was reached in 58.8%, whereas 29.4% experienced a 30–50% of improvement. Mean frequency of headache days dropped from 28 to 12 days at week 24 (*p* = .0001). Randomized controlled trials are needed to confirm these encouraging results.

Randomized clinical trials have indicated that melatonin may be effective for the preventive treatment of CH, with a daily dose of 10 mg [185] and migraine, with a dose of 3 mg [186].

Anti CGRP monoclonal antibodies (mAbs) are effective in migraine prophylaxis [135–138] and the anti CGRP receptor mAbs erenumab is now approved by Food and Drug Administration (FDA) [187]. Ongoing trials (NCT02964338, NCT02797951, NCT02397473, NCT02438826) are investigating the efficacy of anti CGRP mAbs in CH. Recently, an Eli Lilly press release announced that a phase 3 study (NCT02797951) showed that galcanezumab reduced weekly attacks in episodic but not CCH patients [188].

The efficacy of anti CGRP monoclonal antibodies and greater occipital nerve (GON) blockade in both migraine and CH indicates that the activation of the trigeminovascular system (with consequent release of CGRP) and the TCC is a key mechanism involved in the pathogenesis of both migraine and CH. Furthermore, the good response to oral corticosteroids as a transitional treatment may indicate that they may reduce the neurogenic inflammation induced by the activation of the trigeminovascular system in both diseases. The efficacy of melatonin in the prophylactic therapy for both migraine and CH points towards a pathogenetic role for the hypothalamus and the circadian rhythm regulation system in both migraine and CH. The pharmacological effect of verapamil is probably due to the interactions with muscarinic, serotoninergic and dopaminergic receptors, the inhibition of presynaptic adrenergic receptors (with a consequent increase in noradrenaline release) and the modulation of pain pathways. Its efficacy in both migraine and CH could be due to the modulation of brainstem circuitries, the rebalancing of autonomic system and the restoration of the pain control system [189].

In conclusion, even though the first line strategies for migraine and CH treatment seem to be quite different, most of the drugs used for CH prophylaxis also demonstrated a certain degree of efficacy in migraine prophylaxis, showing that migraine and CH, even with their clinical differences may share some of their basic pathophysiological mechanisms.

### Neuromodulation

Invasive neuromodulatory procedures comprise stimulation of the central nervous system, hypothalamic deep brain stimulation (hDBS) and of the peripheral nerves (occipital nerve stimulation, ONS; SPG). Non-invasive variants comprise vagus nerve stimulation (VNS), supraorbital nerve stimulation (SNS), rTMS and transcranial direct current stimulation (tDCS).

The rationale for the use of hDBS is an increased blood flow in the posterior hypothalamus during cluster [74] and migraine attacks [190], which was interpreted as neuronal activation of that brain area. hDBS has been shown to be highly effective in CH, with significant reduction of attack frequency and with the ability to change disease course [22, 191–193]. Although the treatment effects seem clinically equal, the side effects of the more invasive hDBS treatment are to be considered [194]. So far, there is no evidence to support the use of hDBS in CM.

The basis for the use of ONS in headaches came from animal studies showing the convergence of cervical, somatic and dural afferents on second order nociceptors in the trigeminocervical complex [195, 196]. More or less all these structures are involved in the pathophysiology of CM and CH. For ONS, to date, 3 RCTs have been performed in CM [197–199], and their outcome is overall disappointing. For CH multiple isolated reports, case series, small cohort studies and observational studies suggested a 50% improvement in headache frequency or intensity with ONS [200, 201].

The SPG is a large extracranial parasympathetic ganglion located in the pterygopalatine fossa. Post-ganglionic parasympathetic fibers from the SPG innervate facial structures such as the salivary and lacrimal glands, the nasopharyngeal mucosa and the cerebral and meningeal blood vessels [202]. Mainly all these structures are involved in the pathophysiology of CH and partially also in CM. SPG electrical stimulation via an implantable device was proven for effective in a multicentre randomised, double-blind and sham-controlled trial in refractory CCH [203]. Full stimulation of the SPG versus sham stimulation resulted in a significant pain relief (67%) and a significant reduction in attack frequency (34%) [203]. Only anecdotal cases have been reported for migraine treatment with SPG, usually reserved for cases of refractory migraine [204]. SPG has been targeted also with blockade via bupivacaine, which showed, in CM, a sustained reduction of headache frequency in a double-blind, parallel-arm, placebo-controlled, randomized pilot study [205].

VNS has been shown to be effective in both migraine and CH. Indeed, in small open-label single-arm studies, VNS had good migraine abortive effect, with 43 to 65% of patients obtaining pain relief [206, 207]. The recent multicenter, double-blind, randomized, sham-controlled PRESTO trial confirmed VNS effective as abortive treatment for migraine attacks, with consistent therapeutic benefit compared to sham stimulation [208]. In the EVENT trial, a double-blind sham-controlled study on migraine prevention, though not reaching the primary outcome, VNS led to a slight reduction in migraine frequency [209].

CH patients can also benefit from VNS. In an open-label, prospective, randomized study, a significant reduction in weekly attack frequency was observed among patients with CCH receiving VNS plus standard of care compared to standard of care alone [210, 211]. Moreover, VNS has been shown to be cost-effective, providing economic benefits as an adjunct treatment to standard of care in CCH [212].

rTMS has effect as prophylactic treatment in migraine with aura. In a sham controlled randomized trial, single pulse rTMS has been shown to increase in freedom from pain after 2 h when applied early in the treatment of migraine with aura, with substantial benefit for up to 48 h after treatment [213] Although cortical excitability has been implicated in CH [82], to date few data exist on rTMS in CH.

In migraine prevention, SNS has been extensively studied and shown to provide a significant reduction of migraine days compared to sham stimulation [214, 215]. On the contrary, SNS in CH has been poorly investigated, and only isolated reports of possible positive neuromodulation among CH are available [216].

Overall, few data still exist on neuromodulation strategies in headache disorders. Nevertheless, data from randomised controlled trials seem to suggest safety and effectiveness in both migraine and CH (see Table 6), supporting the concept that these two diseases, despite their differences, might share pathophysiological mechanisms. The common denominator might be the hyperexcitability of brain network, progressive changes in nociceptive thresholds and subsequent central sensitization. For CCH, SPG [217, 218] or ONS [197, 219], given the risk/benefit profile of the intervention, might be considered before hDBS. In migraine VNS might be considered as an abortive effective treatment, also able to spare symptomatic drugs. For patients with CM the use of ONS, as well as the application of the non-invasive VNS, tDCS, rTMS, cannot be recommended so far, given the poor amount of controlled data.

**Table 6:** Efficacy of neuromodulation strategies in migraine and cluster headache

Intervention	Migraine	Cluster headache
Deep brain stimulation	Isolated reports, no consistent data	no RCT, available case series show significant reduction in attack frequency but with consistent side effects [22, 117–119]
Occipital nerve stimulation	conflicting results from 3 RCT [197–199]	no RCT, case series show 50% improvement in frequency and intensity [200, 201]
Sphenopalatine ganglion stimulation	no RCT, only anecdotal case reports available	RCT shows SPG electrical stimulation is effective in reducing intensity and frequency in refractory chronic cluster headache [203]
Vagus nerve stimulation	RCT shows effective as abortive treatment [208], slight benefit on migraine frequency in prophylaxis [209]	Conflicting results from RCT, more effective on episodic CH than refractory chronic CH
Transcranial magnetic stimulation	RCT shows benefit on migraine with aura [213]	no RCT, no systematic reports available
Supraorbital nerve stimulation	significant reduction in migraine frequency [214, 215]	no RCT, only isolated reports available, possible positive effect [216]

## Conclusions

Migraine and CH show remarkable similarities with common triggers [22, 114], efficacy of triptans [220, 221], anti CGRP monoclonal antibodies [135–138, 188] and neuromodulation [222]. These observations raise an important question on possible shared pathophysiological mechanisms. The central denominator in both diseases may be the trigeminovascular pathway, alteration in hypothalamic activity and functional changes in hypothalamic–brainstem connectivity. A key signalling molecule, CGRP, is involved in migraine and CH [223, 224]. The importance of the pituitary adenylate-cyclase activating peptide (PACAP) is well established in migraine [140] and an ongoing phase 2 study is testing the efficacy of a PAC1 receptor antibody for migraine prevention [225]. Future studies will show whether migraine and CH shares the involvement of PACAP signalling in pathophysiology.

## Abbreviations

CACNA1A: calcium voltage-gated channel subunit alpha1 A
CCH: chronic cluster headache
cGMP: cyclic guanosine monophosphate
cGMP: cyclic guanosine monophosphate
CGRP: calcitonin gene-related peptide
CH: cluster headache
CM: chronic migraine
FDA: Food and Drug Administration
fMRI: functional magnetic resonance imaging
GON: greater occipital nerve
GTN: glyceryl trinitrate
HCRTR2: hypocretin receptor 2
hDBS: hypothalamic deep brain stimulation
ICHD 3: International Classification of Headache Disorders 3rd edition
LC: locus coeruleus
MwoA: migraine without aura
NO: nitric oxide
NOS: nitric oxide synthase
ONS: occipital nerve stimulation
PAC1: pituitary adenylate cyclase receptor 1
PACAP: pituitary adenylate-cyclase activating peptide
PAG: periequiductal grey
PER3: period circadian regulator 3
PET: positron emission tomography
PREEMPT: Phase III REsearch Evaluating Migraine Prophylaxis Therapy
rTMS: repetitive transcranial magnetic stimulation
rTMS: repetitive transcranial magnetic stimulation
SCN1A: sodium channel 1 A
SNS: supraorbital nerve stimulation
SPG: sphenopalatine ganglion
SuS: superior salivatory nucleus
TCC: trigeminal cervical complex
tDCS: transcranial direct current stimulation
TG: trigeminal ganglion
TNC: Trigeminal nucleus caudalis
VEP: visual evoked potentials
VNS: vagus nerve stimulation
